# Evolution of Bombesin Conjugates for Targeted PET Imaging of Tumors

**DOI:** 10.1371/journal.pone.0044046

**Published:** 2012-09-14

**Authors:** Hanwen Zhang, Keelara Abiraj, Daniel L. J. Thorek, Beatrice Waser, Peter M. Smith-Jones, Michael Honer, Jean Claude Reubi, Helmut R. Maecke

**Affiliations:** 1 Division of Radiological Chemistry, Department of Radiology, University Hospital Basel, Basel, Switzerland; 2 Department of Radiology, Memorial Sloan-Kettering Cancer Center, New York, New York, United States of America; 3 Translational Research Sciences, Pharma Research and Early Development (pRED), F Hoffmann-La Roche Ltd, Basel, Switzerland; 4 Division of Cell Biology and Experimental Cancer Research, Institute of Pathology, University of Berne, Berne, Switzerland; 5 Department of Nuclear Medicine, University Hospital Freiburg, Freiburg, Germany; Genentech, United States of America

## Abstract

Bombesin receptors are under intense investigation as molecular targets since they are overexpressed in several prevalent solid tumors. We rationally designed and synthesized a series of modified bombesin (BN) peptide analogs to study the influence of charge and spacers at the N-terminus, as well as amino acid substitutions, on both receptor binding affinity and pharmacokinetics. This enabled development of a novel ^64/67^Cu-labeled BN peptide for PET imaging and targeted radiotherapy of BN receptor-positive tumors. Our results show that N-terminally positively charged peptide ligands had significantly higher affinity to human gastrin releasing peptide receptor (GRPr) than negatively charged or uncharged ligands (IC_50_: 3.2±0.5 vs 26.3±3.5 vs 41.5±2.5 nM). The replacement of Nle^14^ by Met, and deletion of D-Tyr^6^, further resulted in 8-fold higher affinity. Contrary to significant changes to human GRPr binding, modifications at the N-terminal and at the 6^th^, 11^th^, and 14^th^ position of BN induced only slight influences on affinity to mouse GRPr. [Cu^II^]-CPTA-[βAla^11^] BN(7–14) ([Cu^II^]-BZH7) showed the highest internalization rate into PC-3 cells with relatively slow efflux because of its subnanomolar affinity to GRPr. Interestingly, [^64/67^Cu]-BZH7 also displayed similar affinities to the other 2 human BN receptor subtypes. *In vivo* studies showed that [^64/67^Cu]-BZH7 had a high accumulation in PC-3 xenografts and allowed for clear-cut visualization of the tumor in PET imaging. In addition, a CPTA-glycine derivative, forming a hippurane-type spacer, enhanced kidney clearance of the radiotracer. These data indicate that the species variation of BN receptor plays an important role in screening radiolabeled BN. As well, the positive charge from the metallated complex at the N-terminal significantly increases affinity to human GRPr. Application of these observations enabled the novel ligand [^64/67^Cu]-BZH7 to clearly visualize PC-3 tumors in vivo. This study provides a strong starting point for optimizing radiopeptides for targeting carcinomas that express any of the BN receptor subtypes.

## Introduction

In recent years, bombesin (BN) receptors have attracted interest as molecular targets for imaging and therapy pertaining to the fact that all three BN receptor subtypes are overexpressed in many human tumor types [1]. For example, gastrin releasing peptide receptor (GRPr) has been shown to be overexpressed in prostate [2], [3], breast [4], small cell lung cancer [5] and gastrointestinal stromal tumors [6]. Prostate cancer has been traditionally among the most difficult malignancies to image due to its multifocal nature, demanding imaging solutions that have high sensitivity and good resolution. Radiolabeled BN-based peptides have significant potential as agents for preoperative tumor localization, assessment of lymph node involvement, staging of disease and possibly for therapeutic monitoring of prostate cancer. As such, a number of radiolabeled BN peptide analogs have been developed as targeting vectors for imaging and radionuclide therapy of GRPr positive tumors [7]–[13].

Clinical studies with ^99m^Tc- and ^68^Ga-labeled BN-based peptides have been reported for the imaging of metastasized prostate, breast and gastrointestinal stromal tumors [10], [14]–[16]. A potent BN agonist based peptide labeled with ^177^Lu has been studied in phase 1 clinical trials [10], [17]. More recently, preclinical studies demonstrated that radiolabeled antagonist based BN peptides might even be superior as targeting vectors compared to agonist peptides [18]–[21]. Despite these advances, the limitation imposed by peptide pharmacokinetics with respect to binding and clearance demonstrates that significant improvements of these radiolabeled probes are still required.

PET (Positron Emission Tomography) is a powerful diagnostic imaging modality that enables tomographic, whole body, high sensitivity and quantitative imaging of the distribution of positron emitter-labeled molecules, such as peptides. ^68^Ga-labeled peptides have been extensively studied and effectively implemented in the clinical setting [22]. On the other hand, copper-64 is an interesting radionuclide as it is both a positron- (17.8%, E_β_^+^_max_ = 656 keV) and a β^–^emitter (39.6%, E_β_^−^_max_ = 573 keV) with a half-life of 12.7 h. Long-lived isotopes such as copper-64 may therefore provide the ability to visualize the anatomy of interest after unbound probe has been cleared from nearby structures, such as the bladder. This has the potential to improve detection of disease [23]. Several ^64^Cu-labeled BN analogs have been evaluated as PET tracers targeting GRP receptor positive tumors [24].

In the present study, a series of BN peptides were synthesized and conjugated to DOTA (1,4,7,10-tetraazacyclododecane-1,4,7,10-tetraacetic acid) and CPTA (4-(1,4,8,11-tetraazacyclotetradec-1-yl)-methyl benzoic acid) chelator for labeling with radiocopper. We had several goals in mind when designing this study. Initially, we sought to study the influence of charge at the *N*-terminus of the radiopeptides on their pharmacologic and biologic properties. Earlier work has suggested that the replacement of a tetraamine ligand (antagonistic BN analog) for ^99 m^Tc-labeling [25] by negatively charged DTPA (diethylenetriaminepentaacetic acid) caused affinity drop by a factor of approximately 10^3^ (Reubi, Schmitt, Maecke, unpublished results). A decrease in binding affinity of a BN antagonist was also observed when a tetraamine ligand was replaced by DOTA [26]. We hypothesized that the same effect could also be seen in the class of radiopeptides presented here (agonistic BN analogs). Secondly, the structure of CPTA-ligand allows a modification with glycine to afford a hippurane-like structural spacer. This structure modification was studied here to evaluate its effect on kidney clearance.

In addition, we and others have recently observed that species-specific differences may be of significant importance in the evaluation of bombesin receptor ligands [27]. We therefore studied modifications of BN(7–14) with regard to amino acid substitution at several positions. The final goal of our research is to develop BN-based potent conjugates for labeling with ^64^Cu (^67^Cu: t_1/2_ = 61 h, 100%, E_β_^−^_max_ = 577 keV, Eγ = 185 keV), which could be used in imaging and potentially in radionuclide therapy of BN receptor-positive tumors. As CPTA allows for high labeling yields under very mild labeling conditions, it has been chosen as a bifunctional chelator for the labeling of monoclonal antibodies [28], octreotide [29] and CXCR4 (chemokine receptor 4) ligand [30], [31] with radiocopper. On this basis, a series of CPTA/DOTA conjugated BN analogs were constructed for these research goals.

This work is the first to investigate the influence of different charges at the *N*-terminus of BN analogs on binding affinity, of the hippurane-like spacer molecule on kidney clearance, and of a modification at the 6^th^, 11^th^ and 14^th^ position of these (radio)metallobombesin analogs in different species. Together, we show these investigations play an important role towards optimizing radioligands for diagnosis and targeted radionuclide therapy of bombesin receptor-positive tumors.

## Materials and Methods

All chemicals were obtained from commercial sources and used without further purification. ^64^Cu/^67^Cu nuclides were produced for *in vitro* assays and the biodistribution studies at the 72 MeV accelerator of the Paul Scherrer Institute (Villigen, Switzerland) by irradiating ^nat^Zn with protons [32]. For small-animal PET imaging, ^64^Cu was obtained from Washington University in St. Louis. CPTA was synthesized as described previously [33]. Rink amide MBHA resin and all Fmoc-protected amino acids were commercially available from NovaBiochem (Läufelfingen, Switzerland). DOTA-tris(^t^Bu)-ester was purchased from CheMatech (Dijon, France). [^111^In]Cl_3_ was purchased from Covidien plc (Dublin 2, Ireland). Electrospray ionization (ESI) mass spectroscopy was carried out with a Finnigan SSQ 7000 spectrometer, fast atom bombardment (FAB) mass spectroscopy with a VG 70SE spectrometer and MALDI-MS measurement on a Voyager sSTR equipped with an Nd:YAG laser (Applied Biosystems, Framingham, USA). Analytical HPLC was performed on a Hewlett Packard 1050 HPLC system (Waldbronn 2, Germany) with a multiwavelength detector and a flow-through Berthold LB 506 Cl g-detector (Wildbad, Germany) using a Macherey-Nagel Nucleosil 120 C_18_ column (Oensingen, Switzerland). Preparative HPLC was performed on a Metrohm HPLC system LC-CaDI 22–14 (Herisau, Switzerland) with a Macherey-Nagel VP 250/21 Nucleosil 100-5 C_18_ column. Quantitative gamma counting was performed on a COBRA 5003 gamma system well counter from Packard Instruments (Meriden, CT, USA). Solid phase peptide synthesis was performed on a semiautomatic peptide synthesizer commercially available from Rink Combichem (Bubendorf, Switzerland). The PC-3 cell line was obtained from ATCC (Manassas, VA) and cultured in Dulbecco’s minimal essential medium (DMEM) with 10% fetal calf serum (FCS) from BioConcept (Allschwil, Switzerland). Small-animal PET imaging was performed on a R4 microPET scanner (Concorde Microsystems, Knoxville, TN).

### Synthesis

The peptides were synthesized on solid phase using standard Fmoc strategy. The bifunctional chelator CPTA was coupled to the resin-assembled peptide as follows: 6 equivalents CPTA were mixed together with 18 equivalents PyBop, 18 equivalents HoBt and 80 equivalents DIPEA in NMP and immediately incubated with the resin-assembled peptide until the TNBS test was negative (approximately 5 h). DOTA-tris(^t^Bu)-ester was coupled to the *N*-terminus of the peptide on resin as follows: 3 equivalents of DOTA-tris(^t^Bu)-ester, which was pre-activated with 3.3 equivalents of HATU in NMP, was treated with 6 equivalents of DIPEA and immediately incubated with the resin-assembled peptide until the Kaiser test was negative (approximately 4 h).

### Preparation of Metallated Conjugates

The peptides used in the following studies are listed in Table 1. Peptide (0.5 µmol) dissolved in 500 µL 0.5 M ammonium-acetate-buffer (pH 5) was incubated with 1.5 µmol CuCl_2_·2H_2_O pre-dissolved in 0.04 M HCl for 1 h at room temperature, and purified over a SepPak C_18_ cartridge (Waters Corp. Milford, MA) preconditioned with 10 mL ethanol and 10 mL water. The cartridge was eluted with 10 mL water followed by 3 mL methanol resulting in Cu^II^-peptides after evaporation of the methanol. The final product was analyzed with analytical HPLC and MALDI. Using 3 equivalents InCl_3_·5H_2_O, [In^III^]-BZH4 was synthesized at elevated temperature (95°C, 20–25 min) and purified as described above.

**Table 1:** Characteristics of compounds BZH4, BZH5, BZH6, BZH7 and BZH8, and their corresponding cold metallated compounds.

Compound^†^	Calculated MW	Measured MW			RP-HPLC‡ retention time (min)
		ESI (+)	ESI (−)	MALDI	
BZH4	1570.79	1609.1 ([M+K]^+^)	1607.7 ([M+K−H]^−^)	1570.5 ([M+H]^+^)	13.61
BZH5	1415.73	1416.5 ([M+H]^+^)	1414.8 ([M−H]^−^)	1415.7 ([M+H]^+^)	14.54
BZH6	1433.77	1434.1 ([M+H]^+^)	1432.3 ([M−H]^−^)	1433.8 ([M+H]^+^)	12.76
BZH7	1270.59	1270.9 ([M+H]^+^)	1269.7 ([M−H]^−^)	1270.5 ([M+H]^+^)	12.97
BZH8	1327.64	1328.1 ([M+H]^+^)	1326.7 ([M−H]^−^)	1327.8 [M+H]^+^	12.54
[Cu^II^]-BZH4	1632.32	816.5 ([M+2H]^2+^)	814.3 ([M−2H]^2−^)	1632.4 [M+H]^+^	15.28
[In^III^]-BZH4	1682.58	ND	ND	1682.4 [M+H]^+^	14.81
[Cu^II^]-BZH5	1479.27	ND	ND	1477.6 [M+H]^+^	16.20
[Cu^II^]-BZH6	1497.31	ND	ND	1497.7 [M+H]^+^	13.18
[Cu^II^]-BZH7	1334.14	ND	ND	1332.4 [M+H]^+^	13.83
[Cu^II^]-BZH8	1391.19	ND	ND	1392.3 [M+H]^+^	13.32

Note: ^†^MW: molecular weight; BZH4: **DOTA-GABA-[D-Tyr^6^, βAla^11^, Nle^14^] BN(6–14)**; BZH5: **CPTA-[D-Tyr^6^, βAla^11^, Nle^14^] BN(6–14)**; BZH6: **CPTA-[D-Tyr^6^, βAla^11^] BN(6–14)**; BZH7: **CPTA-[βAla^11^] BN(7–14)**; BZH8: **CPTA-[Gly^6^, βAla^11^] BN(6–14)**.

‡ RP HPLC eluents: A = 0.1% TFA in water and B  =  acetonitrile; gradient: 0–20 min, 80%–50% A; 20–21 min, 100% B; 21–24 min, 100% B; 25 min, 80% A.

### Preparation of Radiotracer for *in vitro* and *in vivo* Studies

[^64/67^Cu]-BZH7, denoting a mixture of [^64^Cu]-BZH7 and [^67^Cu]-BZH7, was prepared by dissolving 10 µg of BZH7 (7.5 nmol) in ammonium acetate buffer (300 µL, 0.5 M, pH 5.5); after the addition of ^64/67^CuCl_2_ (about 185 MBq ^64^Cu and 37 MBq ^67^Cu), the solution was incubated at room temperature for 1 h. A 1.5 molar excess of CuCl_2_·2H_2_O was added and incubated again for 0.5 h. Subsequently, radiolabeled peptides were purified utilizing a SepPak C18 cartridge preconditioned with 10 mL methanol and 10 mL water; the cartridge was eluted with 3 mL water, followed by 2 mL ethanol, to afford the pure ^64/67^Cu-labeled ligand. For biodistribution studies, the labeling was performed accordingly without adding cold CuCl_2_·2H_2_O. The solution was prepared for injection by dilution with 0.9% NaCl (0.1% BSA) to afford the radioligand solution. All ^64/67^Cu-labeled conjugates were prepared in the same way. The preparation of [^111^In]-BZH4 was described previously [12]. All radiolabeled peptides were analyzed with HPLC (eluents: A = 0.1% TFA in water and B  =  acetonitrile; gradient: 0–20 min, 80%–50% A; 20–21 min, 100% B; 21–24 min, 100% B; 25 min, 80% A). Pure ^64^Cu was used for small animal PET imaging of PC-3 xenografts.

### Binding Affinity and Receptor Subtype Profile

Using [^125^I-Tyr^4^] BN as a GRP receptor preferring ligand, the IC_50_ values of the ^nat^Cu/^nat^In-labeled peptides were measured by in vitro autoradiography of sections of human prostate cancer tissue overexpressing GRP receptors or mouse pancreas tissue expressing mouse GRP receptors. The prostate cancer tissues originated from samples investigated previously [2] or collected prospectively at the Institute of Pathology of the University of Berne in accordance with international ethical guidelines, including informed written consent and approval by the institutional review board. The binding affinity profile of [Cu^II^]-BZH7 for three bombesin receptor subtypes was determined by using [^125^I-D-Tyr^6^, βAla^11^, Phe^13^, Nle^14^] BN(6–14) as an universal radioligand. The procedures were described in detail previously [34].

### Internalization and Externalization (efflux) Studies

Internalization and externalization experiments were performed in 6-well plates as described previously [12]. Briefly, for internalization studies, approximately 1.3 kBq (0.25 pmol) of radioligand was added to the medium and PC-3 cells, 1 million cells per well, incubated in triplicate for 0.5, 1, 2, 4, and 6 h at 37°C, 5% CO_2_. One hundred and fifty µL of a 5.8 µM BZH2 solution (DOTA-GABA-[D-Tyr^6^, βAla^11^, Thi^13^, Nle^14^] BN(6–14) was used to determine nonspecific internalization. For externalization studies, PC-3 cells were allowed to internalize the radioligands for a period of 2 h at 37°C and were then exposed to 1 mL of culture medium to measure efflux kinetics.

### Serum Stability

The procedures were previously described in details [12]. Briefly, 50 µL, 0.6 nmol ^111^In- or ^64/67^Cu-labeled conjugates were used and incubated with human serum at different time points (0, 1, 4, and 8 h), in triplicate. The HPLC profiles from sample analysis were used to calculate the half-life of disappearance of intact peptide.

### Biodistribution Studies with Mice Bearing PC-3 Tumor

After being brought to the condition of anesthesia with isoflurane in an air/oxygen mixture, female athymic nude mice were implanted subcutaneously with approximately 10 million PC-3 tumor cells, which were freshly expanded in 100 µL sterilized PBS solution. Seven to ten days after inoculation the tumors weighed 60–130 mg. The xenografts were injected via tail vein with 10 pmol radiolabeled peptides (about 0.24 MBq ^64^Cu and 0.05 MBq ^67^Cu), diluted in 0.9% NaCl (0.1% BSA, pH 7.4, total injected volume  =  100 µL). For the determination of non-specific uptake in the tumor or receptor-positive organs, a group of 4 animals were injected with a mixture of 10 pmol radiolabeled peptide/50 µg [Cu^II^]-BZH7 in 0.9% NaCl solution (injected volume 150 µL). Mice were sacrificed at 1, 4 and 24 h, and organs of interest collected, rinsed of excess blood, blotted, weighed and counted in a **γ**-counter. The percentage of injected activity per gram (% IA/g) for each tissue was calculated. The total counts injected per animal were determined by extrapolation from counts of an aliquot taken from the injected solution as a standard. All animal experiments were performed in compliance with the Swiss regulations for animal treatment, as approved by the Federal Veterinary Office (Bundesamt für Veterinärwesen, approval no. 789). Written consent in the form of an official internal document was given.

### Small Animal PET Imaging

Nude female mice with a PC-3 tumor xenograft on the right shoulder were injected with 1.0 nmol 4.6 MBq [^64^Cu]-BZH7 via tail vein. Small-animal PET imaging was performed at 1, 4 and 24 h p.i. using the R4 microPET scanner (Concorde Microsystems, Knoxville, TN), with the tumors centered in the field of view. Mice were maintained at 2% isoflurane/air anesthesia for the duration of the imaging. Data acquisitions were performed for 10 min with an energy window of 250–750 keV and a coincidence-timing window of 6 ns. Analysis of the acquired images was performed using ASIPro software (Siemens Medical Solutions USA, Inc., Malvern, PA).

PET imaging studies were conducted at Memorial Sloan-Kettering Cancer Center (MSKCC). All work was evaluated and approved by the Institutional Animal Care and Utilization Committee (IACUC) of MSKCC (approval no. 08-07-011). Written consent of this protocol was provided by IACUC.

### Statistical Analysis

Data are expressed as mean ± SD, calculated using Microsoft Excel. The Student’s t-test (Origin 6, Microcal Software, Inc., Northampton, MA) was used to determine statistical significance at the 95% confidence level. Values of P<0.05 were considered significantly different.

## Results

### Synthesis and Labeling

**Figure 1:**
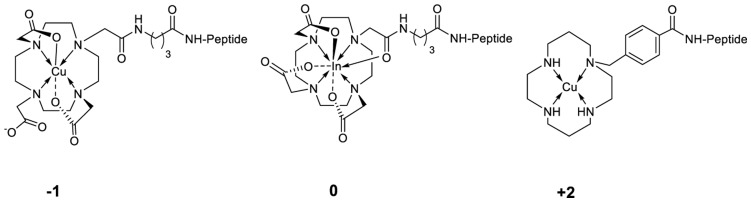
Scheme of metal-complexed conjugates generating different charges at the N-terminus under the condition of pH 7.4.

**Figure 2:**
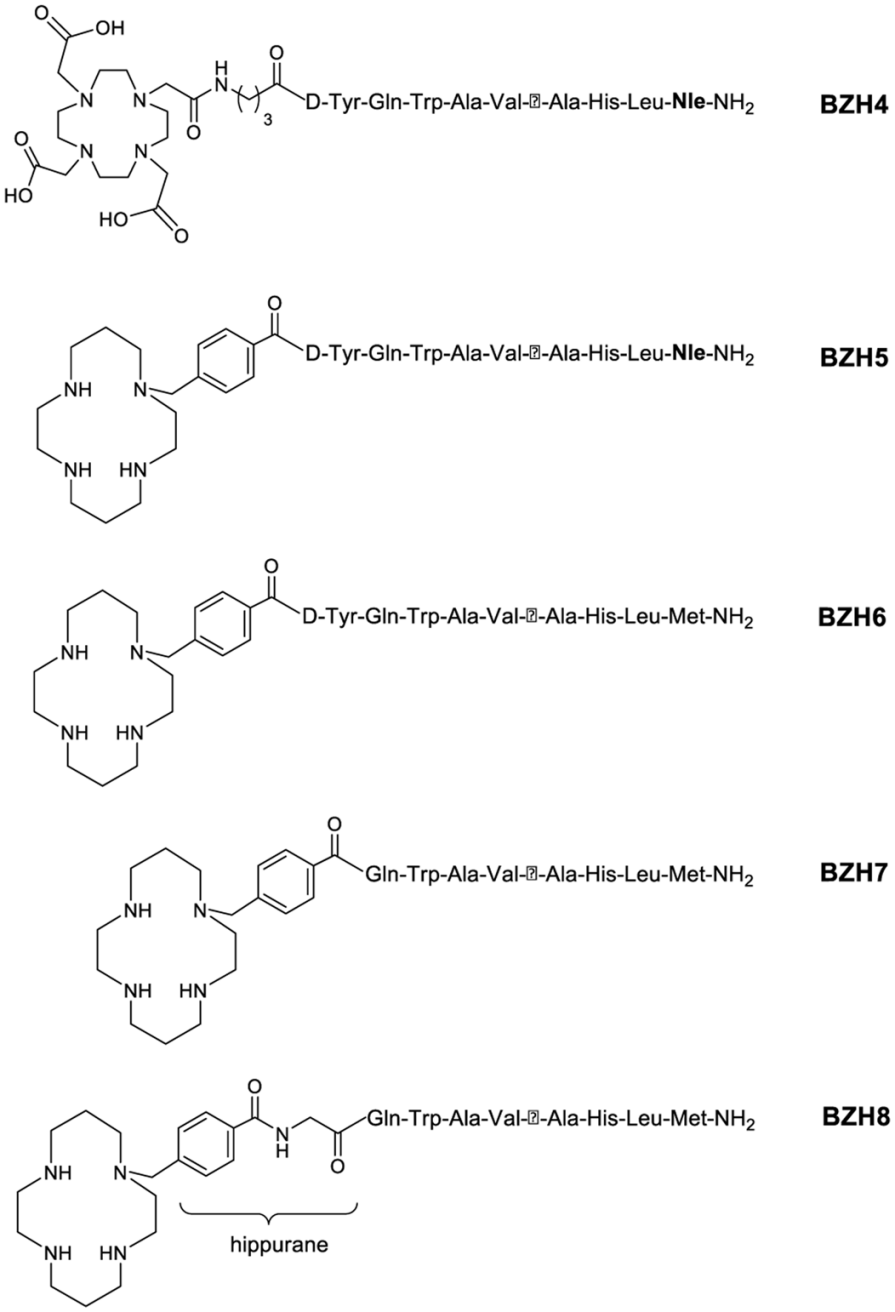
Scheme of modification on BN peptide to optimize affinity unit, form hippurane-like spacer and to functionalize for radiolabeling with ^64/67^Cu and ^111^In.

All conjugates (Table 1, Figures 1 and 2) were synthesized using an Fmoc strategy affording a maximum yield of approximately 30% based on the removal of the first Fmoc group; the purity analyzed by HPLC was ≥97%. BZH4 was labeled with ^111^In at an elevated temperature (95°C) for 20–25 min, and all other conjugates were labeled with ^64/67^Cu at room temperature. In all cases, radiolabeling yields of ≥98% at specific activities of >24 GBq µmol^−1^ were achieved for ^64^Cu, >5 GBq µmol^−1^ for ^67^Cu and >37 GBq µmol^−1^ for [^111^In]-BZH4.

### Receptor Binding Affinity

Competitive binding assays were performed with human GRP receptor-positive cancerous tissue and mouse pancreas tissue (expressing mouse GRP receptor) using [^125^I-Tyr^4^]bombesin as radioligand. Table 2 summarizes the binding affinities of the metallopeptides to GRP receptors of mouse and human origin. The Cu^II^- and In^III^-complexed peptides exhibit a wide dynamic range of binding (high to moderate affinity) to human GRP receptor (0.42±0.13 nM to 41.5±2.5 nM). In contrast, all of these probes display similar high affinity to mouse GRP receptor (0.22±0.07 nM to 1.1±0.33 nM).

**Table 2:** IC_50_ values for displacement of GRP receptor-bound [^125^I-Tyr^4^] BN by increasing concentration of BN analogs.

Code No.	Peptide structure	Charge	GRP receptor	
			human	mouse
[Cu^II^]-BZH4	Cu^II^-DOTA-GABA-[D-Tyr^6^, βAla^11^, Nle^14^] BN(6–14)	−1	26.3±3.5 (3)	1.1±0.3 (3)
[In^III^]-BZH4	In^III^-DOTA-GABA-[D-Tyr^6^, βAla^11^, Nle^14^] BN(6–14)	0	41.5±2.5 (2)	0.8±0.4 (2)
[Cu^II^]-BZH5	Cu^II^-CPTA -[D-Tyr^6^, βAla^11^, Nle^14^] BN(6–14)	+2	3.2±0.5 (3)	0.6±0.2 (3)
[Cu^II^]-BZH6	Cu^II^-CPTA-[D-Tyr^6^, βAla^11^] BN(6–14)	+2	1.0±0.2 (3)	0.8±0.2 (3)
[Cu^II^]-BZH7	Cu^II^-CPTA-[βAla^11^] BN(7–14)	+2	0.42±0.13 (4)	0.22±0.07 (3)
[Cu^II^]-BZH8	Cu^II^-CPTA-[Gly^6^, βAla^11^] BN(6–14)	+2	1.8±0.6 (3)	0.8±0.2 (3)

IC_50_ values (nM ± SD) are in triplicates. Number of independent studies is in brackets.

The most promising peptide, Cu-BZH7, was also studied with respect to BN receptor subtype profiles using human cancerous tissues shown to express predominantly a single bombesin receptor subtype. The peptide showed very high binding affinity to all 3 human BN receptor subtypes (0.27±0.16 nM to NMB-R; 0.30±0.07 nM to GRP-R; 1.4±0.6 nM to BNRS-3).

### Internalization and Efflux Studies

**Figure 3:**
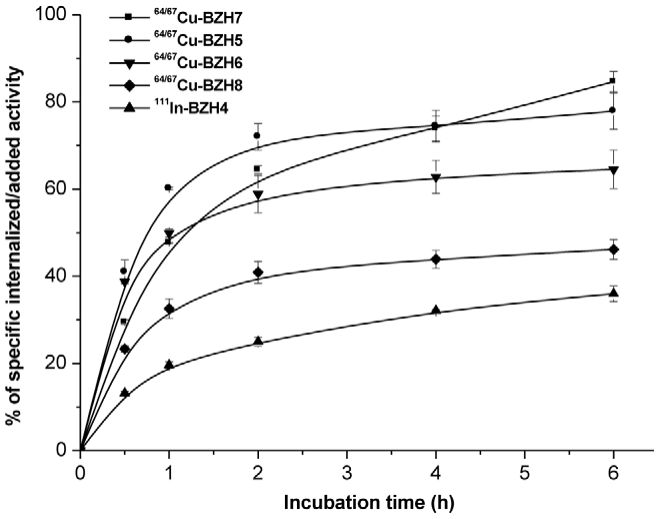
Comparison of the internalization of [^111^In]-BZH4 and [^64/67^Cu]-labeled BZH5, BZH6, BZH7 and BZH8 into PC-3 cells demonstrated both higher affinity of ligand and positive charge at the N-terminus of ligand determined a faster and higher internalization rate in GRP receptor expressing cells. Results from two independent experiments with triplicates in each experiment, expressed as specific internalization.

The internalization kinetics of [^64/67^Cu]-BZH5–8 and [^111^In]-BZH4 in PC-3 cells at 37°C is summarized in Figure 3. All radiopeptides showed specific, receptor-mediated cell uptake. [^64/67^Cu]-BZH5 and [^64/67^Cu]-BZH7 internalized into PC-3 cells very efficiently, reaching about 80% of total activity added to a well containing 1 million cells within 6 h. [^64/67^Cu]-BZH6, [^64/67^Cu]-BZH8 and [^111^In]-BZH4 had internalization rates of 64%, 46%, and 36% at 6 h under the same experimental conditions. Internalization was almost completely blocked (nonspecific internalization was <1% of the added activity) in presence of 0.57 µM unlabeled DOTA-GABA-[D-Tyr^6^, βAla^11^, Thi^13^, Nle^14^] BN(6–14). Surface-bound peptide (radioactivity removable by acid wash) was <3% of the added radiopeptide at each time point.

**Figure 4:**
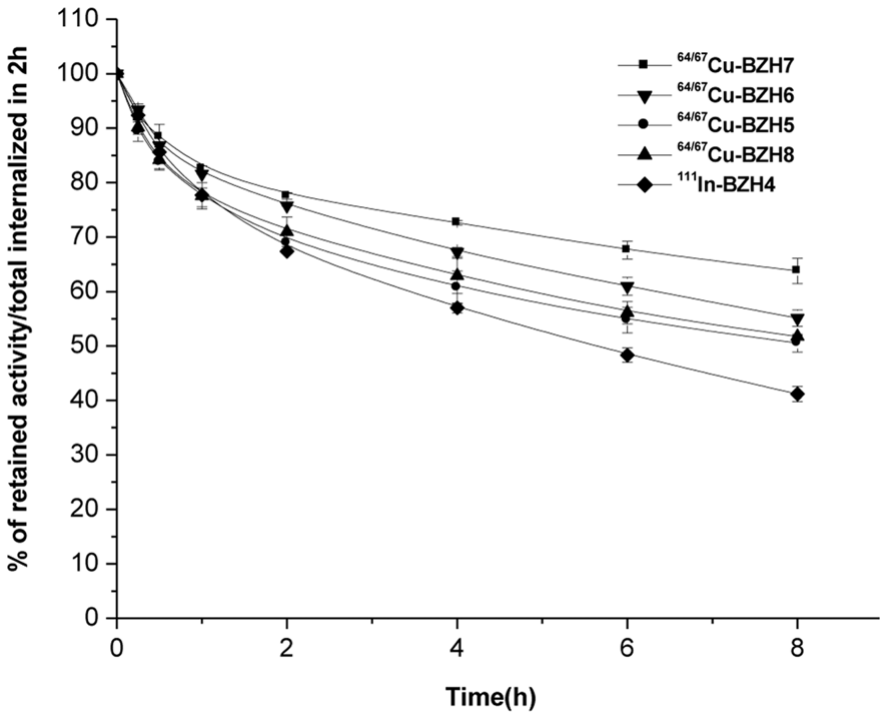
Comparison of the externalization of [^111^In]-BZH4, [^64/67^Cu]-labeled BZH7, BZH5, BZH6 and BZH8 from PC-3 cells showed [^64/67^Cu]-labeled BZH7 had a relatively slow efflux as a result of its high affinity. Result from two independent experiments with triplicates in each experiment.

The efflux kinetics were studied in PC-3 cells that were exposed to radioligand for 2 h as described for internalization, followed by an acid wash, and then incubated with medium (1% FCS). The results are summarized in Figure 4. Upon 8 h incubation, 36% of the pre-internalized [^64/67^Cu]-BZH7, 45% of [^64/67^Cu]-BZH6, 51% and 52% of [^64/67^Cu]-BZH5 and [^64^Cu]- BZH8, and 60% of [^111^In]-BZH4 were washed out from the PC-3 cells.

To identify the composition of the externalized peptides, [^111^In]-BZH4 (^111^In-DOTA-GABA-D-Tyr-Gln-Trp-Ala-Val-βAla-His-Leu-Nle-NH_2_) was used as a surrogate peptide with high specific activity, required for metabolic studies. Upon 2 h of internalization and acid wash, the externalized radioactivity after 2 h incubation already consisted of approximately 84% metabolites (^111^In-DOTA: 14%; ^111^In-DOTA-GABA-D-Tyr-Gln: main peak, 64%; ^111^In-DOTA-GABA-D-Tyr-Gln-Trp-Ala-Val-βAla: 6%) and 16% intact peptide. These results indicate that the internalized ligands can be decomposed quickly in the targeted cells; and their retention in cells is determined mainly by their metabolic stability.

### Stability in Human Serum

Serum stability was studied to determine the half-life of disappearance of intact peptides in serum (Table 3). There was less than 3% of radiometal transfer to serum proteins during serum incubation studies. Using the equation of A = A_0_*exp(-k1*t), the half-lives (t_1/2_) of disappearance of intact peptides in serum were calculated [12]; they varied between 0.55 and 5.1 h. The N-terminally attached chelate, [^111^In]-DOTA in [^111^In]-BZH4 and [^64/67^Cu]-CPTA in [^64/67^Cu]-BZH5 did not influence metabolic stability in serum (0.61±0.11 h versus 0.55±0.11 h). The replacement of methionine ([^64/67^Cu]-BZH5) by norleucine ([^64/67^Cu]-BZH6) increased the t_1/2_ value by 40%. The t_1/2_ values of [^64/67^Cu]-BZH7 and [^64/67^Cu]-BZH8 were 5.1±1.7 h and 4.5±1.2 h, respectively.

**Table 3:** Kinetic metabolic degradation of ^111^In/^64/67^Cu-labeled bombesin analogs, which was calculated according to the equation of A = A_0_*exp(-k_1_*t).

Conjugates	k_1_ (h^−1^)	T_1/2_ (h)
[^111^In]-BZH4	1.14±0.26	0.61±0.11
[^64/67^Cu]-BZH5	1.26±0.31	0.55±0.11
[^64/67^Cu]-BZH6	0.756±0.168	0.92±0.17
[^64/67^Cu]-BZH7	0.137±0.066	5.1±1.7
[^64/67^Cu]-BZH8	0.154±0.054	4.5±1.2

### Biodistribution Studies

^64/67^Cu-labeled peptides were validated with athymic nude mice bearing PC-3 tumor xenograft. Results are presented in Tables 4 and 5 as percentage of injected activity per gram of tissue (%IA/g).

**Table 4:** Comparison of biodistribution of [^64/67^Cu]-BZH5, [^64/67^Cu]-BZH6, [^64/67^Cu]-BZH7, and [^64/67^Cu]-BZH8 at 4 h p.i. in PC-3 tumor-bearing nude mice.

Site	[^64/67^Cu]-BZH5	[^64/67^Cu]-BZH6	[^64/67^Cu]-BZH7	[^64/67^Cu]-BZH8
Blood	0.182±0.011	0.175±0.021	0.154±0.033	0.142±0.004
Muscle	0.087±0.006	0.109±0.025	0.123±0.032	0.064±0.011
Kidneys	4.59±0.65	5.84±0.55	6.87±1.16	4.97±0.46
Adrenals	25.7±2.2	27.3±3.7	30.8±4.1	25.3±1.3
Pancreas	34.9±1.6	58.0±3.9	57.2±2.7	36.4±3.4
Spleen	2.66±0.06	3.67±0.25	3.77±0.62	2.93±0.40
Stomach	1.81±0.17	3.42±0.82	3.76±0.48	2.81±0.29
Bowel	4.62±0.23	8.19±1.06	7.25±0.74	6.44±0.21
Liver	10.78±1.32	10.01±1.03	7.31±0.87	8.51±0.44
Lung	0.48±0.16	0.72±0.12	0.67±0.13	0.78±0.18
Heart	0.25±0.04	0.35±0.08	0.22±0.05	0.20±0.02
Bone	0.34±0.04	1.05±0.23	0.90±0.16	0.49±0.07
Tumor	3.47±0.15	5.05±0.46	6.63±0.80	4.29±0.70
Tumor to normal tissue radioactivity ratio				
Tumor/Blood	19	29	43	30
Tumor/Muscle	40	46	54	67
Tumor/Liver	0.3	0.5	0.9	0.5
Tumor/Kidney	0.8	0.9	1.0	0.9

Results are the mean (%IA/g) of groups of eight or four animals.

**Table 5:** Kinetic biodistribution of [^64/67^Cu]-BZH7 in PC-3 tumor-bearing nude mice.

Site	1 h	4 h	24 h	4 h, Blocked
Blood	0.461±0.078	0.154±0.033	0.102±0.010	0.149±0.013
Muscle	0.539±0.384	0.123±0.032	0.072±0.018	0.082±0.002
Kidneys	10.4±1.2	6.87±1.16	2.48±0.25	2.44±0.10
Adrenals	36.5±5.7	30.8±4.1	5.29±0.36	1.39±0.06
Pancreas	81.3±15.2	57.2±2.7	18.7±1.6	1.30±0.08
Spleen	5.03±0.40	3.77±0.62	2.15±0.21	0.88±0.05
Stomach	3.98±0.32	3.76±0.48	2.23±0.39	0.51±0.07
Bowel	10.8±1.42	7.25±0.74	5.14±0.20	0.63±0.16
Liver	9.89±0.89	7.31±0.87	3.01±0.47	8.90±0.43
Lung	1.01±1.42	0.67±0.13	0.64±0.31	0.58±0.02
Heart	0.34±0.05	0.22±0.05	0.21±0.03	0.14±0.01
Bone	1.16±0.20	0.90±0.16	0.43±0.10	0.21±0.06
Tumor	11.2±1.5	6.63±0.80	4.14±0.55	0.71±0.08
Tumor to normal tissue radioactivity ratio				
Tumor/Blood	24	43	41	
Tumor/Muscle	21	54	58	
Tumor/Liver	1.1	0.9	1.4	
Tumor/Kidney	1.1	1.0	1.7	

Results are the mean (%IA/g) of groups of eight or four animals. The 4 h time point data is reproduced from Table 4.

All [^64/67^Cu]-CPTA conjugated ligands display a similar rapid blood clearance from PC-3 tumor-bearing mice, varying between 0.142%IA/g to 0.182%IA/g at 4 h (Table 4). There was also a rapid clearance from GRP-R-negative organs except kidneys and liver. High uptakes were observed both in human prostate tumor xenografts and mouse GRP-R-positive organs; e.g. at 4 h p.i., tumor uptake was 3.47±0.15%IA/g for [^64/67^Cu]-BZH5, 5.05±0.46%IA/g for [^64/67^Cu]-BZH6, 6.63±0.80%IA/g for [^64/67^Cu]-BZH7, and 4.29±0.70%IA/g for [^64/67^Cu]-BZH8. Their uptake in the pancreas was 34.9±1.6%IA/g, 58.0±3.9%IA/g, 57.2±2.7%IA/g, and 36.4±3.4%IA/g, respectively. Of these four conjugates, [^64/67^Cu]-BZH7 showed the lowest liver uptake.

*In vivo* competition experiments (Table 5) using 50 µg [Cu^II^]-BZH7 co-injected with [^64/67^Cu]-BZH7 resulted in a >89% reduction of tumor uptake and also a reduction of uptake in GRP-R-positive organs, e.g. >97% in pancreas, 96% in adrenals, 91% in bowel, 84% in stomach, 76% in spleen and bone. These uptake values through co-injected peptide blocking were all significantly decreased (P<0.05). The co-injection of [Cu^II^]-BZH7 led to a somewhat increased liver uptake, whereas the uptake in kidneys was partially blocked. The injection of a blocking dose had no significant influence on the uptake in other non-target organs (p>0.05). Due to rapid clearance of the peptides, high tumor-to-background ratios were obtained (Tables 4 and 5). [^64/67^Cu]-BZH7 showed the greatest tumor-to-background and tumor-to-liver ratios. The kinetics of [^64/67^Cu]-BZH7 (Table 5) showed a high initial accumulation in the tumor (11.2±1.5%IA/g at 1 h p.i.), followed by a decreased uptake to 6.63±0.80%IA/g at 4 h and to 4.14±0.55%IA/g at 24 h p.i., indicating a rapid initial wash out. The ratios between tumor and background (blood and muscle) were >20 at 1 h p.i. and increased to >40 at 24 h. The ratios between tumor and liver or kidney also increased slightly from 1 to 24 h.

**Figure 5:**
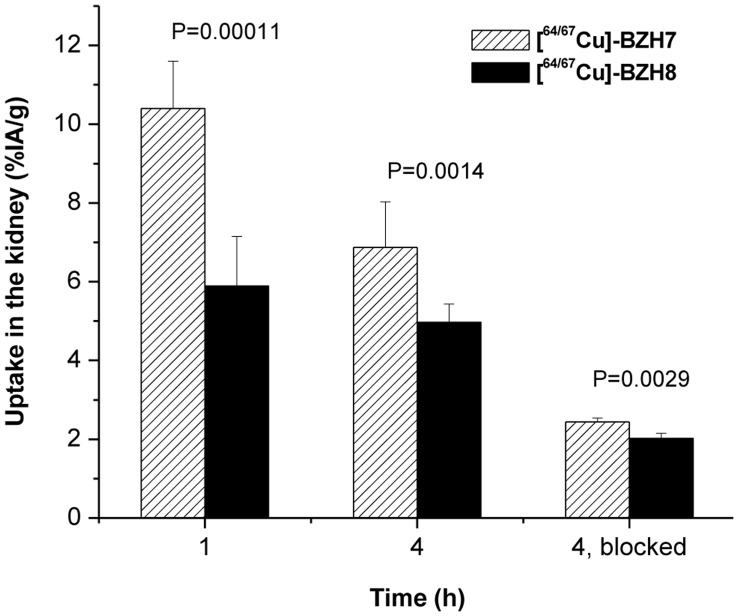
CPTA-glycine derivative, forming a hippurane-type spacer molecule, demonstrates a significantly decreased uptake in kidney at 1, 4 h p.i. and 4 h p.i. blocked with an excess of BN peptide.

The CPTA-glycine derivative, forming hippurane-type spacer (Figure 2) reduced the retention of radioactivity in kidneys (Table 4, Figure 5). The uptake of [^64/67^Cu]-BZH7 and [^64/67^Cu]-BZH8 in kidney differed by a factor of 1.8 at 1 h p.i. (P<0.001) and of about 1.4 at 4 h (6.9±1.2%IA/g versus 5.0±0.5%IA/g, P = 1.4×10^−3^). Under the condition of excessive Cu-BZH7, the uptake of [^64/67^Cu]-BZH8 in kidney was also lower significantly than that of [^64/67^Cu]-BZH7 (2.02±0.13%IA/g versus 2.44±0.10%IA/g, (P = 2.9×10^−3^).

### Small Animal PET Imaging

**Figure 6:**
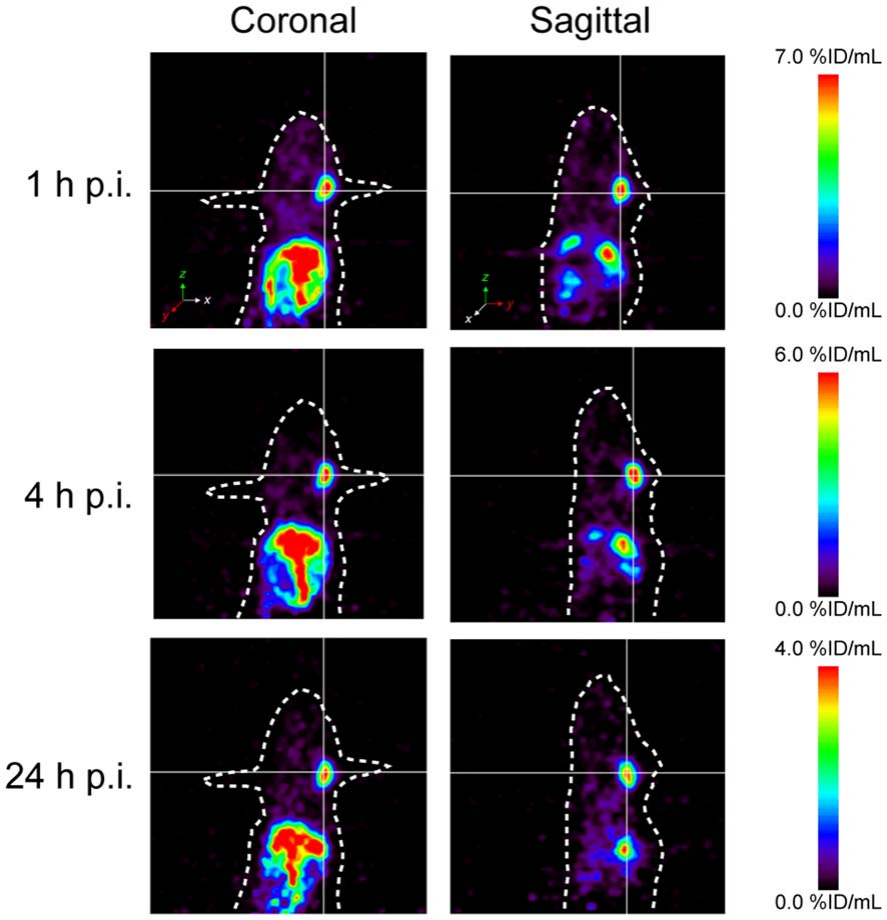
PET imaging of a PC-3 tumor bearing nude mice with 4.6 MBq [^64^Cu]-BZH7 at 1, 4 and 24 h post-injection. PC-3 tumor inoculated in mouse shoulder is visualized and can be clearly distinguished from liver, kidneys and adjacent tissue.

Whole-body PET scanning of PC-3 tumor bearing mice was performed with [^64^Cu]-BZH7, as shown in Figure 6. The PC-3 tumor on the right shoulder was clearly visualized at 1, 4, and 24 h p.i.; and it could be distinguished well from other organs. [^64^Cu]-BZH7 displayed a high uptake in gut. Pancreas (mouse GRP receptor positive organ), liver, kidneys and the urinary bladder also displayed some activity. The low uptake of [^64^Cu]-BZH7 was found in the blood pool, which resulted in a high tumor-to-background ratio. There was a negligible hepatobiliary elimination of the radiopeptide, as implied by a low accumulation in intestine.

## Discussion

This study describes synthesis, characterization and evaluation of radiocopper-chelated BN analogs for PET imaging. [^64^Cu]-BZH7 has shown to be a potential candidate for further development as PET tracer. This is due to its high affinity to NMB, GRP and BB3 receptors and a high rate of internalization into GRP receptor expressing cells.

### Binding Affinity

Our earlier unpublished work has suggested that charge differences at the N-terminus of BN targeting peptides could effect drastic changes on receptor binding. Here, charges were introduced at the *N*-terminus of BN peptides in a dual-purpose strategy to change the charge and functionalize the peptides for radiolabeling by attaching different metal-chelate complexes. Compared to negative- or neutral charges, positive charge was found to significantly improve IC_50_ values of BN peptides by a factor of 8.2 and 13.0, respectively, for human GRP receptor. This result indicates that bifunctional chelators which serve to introduce an N-terminal positive charge may be a good choice for the development of copper-64 labeled BN analogs. These findings explain why the positively charged [^99 m^Tc]-labeled bombesin analog [25] displays high affinity to GRP receptor and high accumulation in PC-3 tumors whereas non-positively charged analogs, such as ^64^Cu-labeled DOTA-Aoc-BN(7–14) [35] or ^111^In-labeled DOTA-[Lys^3^] BN [36] show low affinities.

In contrast to the effect of these charge differences on binding to human GRP receptor, the three differently charged peptides (Table 2 and Figure 1) show only slight differences in the affinity to mouse GRP receptor. This surprising result indicates that mouse GRP receptor is not sensitive to the modifications at the *N*-terminus. Further, we have shown that the other BN peptide modifications performed in this study are also of little effect. This is a strong indication that the mouse pancreas, which is commonly used to screen the performance of new BN based ligands, may in fact not be a good predictor of probe utility.

The substitution of methionine by norleucine is expected to prevent radio-oxidation of thioether group and potentially has a negligible effect on binding affinity to GRP receptor. We previously found that the replacement of methionine by norleucine does not change IC_50_ values of our panbombesin analogs (norleucine in the 14^th^ position). The data in Table 2 show that the substitution of Met^14^ ([Cu^II^]-BZH6) by Nle^14^ ([Cu^II^]-BZH5) decreased binding affinity to human GRP receptors from 1.0±0.2 nM to 3.2±0.5 nM whereas it had no significant influence on their affinities to mouse GRP receptors (0.8±0.2 nM versus 0.6±0.2 nM).

[Cu^II^]-BZH7 showed the highest affinity to human and mouse GRP receptors (0.42±0.13 nM versus 0.22±0.07 nM) that we have found in more than 100 BN analogs tested in our lab. By introducing Gly (D-Tyr) between CPTA and [βAla^11^] BN(7–14) the binding affinity toward human GRP receptor dropped by a factor of 4.3 (2.4) and to mouse GRP receptor by a factor of 3.6 (3.6), respectively, indicating that the insertion of glycine or D-Tyrosine does not improve binding affinity when there is a metal-complexed bifunctional chelator at the *N*-terminus. This is not the case with D-Phe or D-Tyr in the sequence of [D-Tyr^6^, βAla^11^, Phe^13^, Nle^14^] BN(6–14) [37], [38] or BN(6–14) [39] which have been shown to be important to maintain high binding affinity.

[Cu^II^]-BZH7 (cyclam-(4-methylbenzoyl)-[βAla^11^] BN(7–14*)*) displayed very high affinities to all 3 human BN receptor subtypes, as does the known pan-bombesin ligand [D-Tyr^6^, βAla^11^, Phe^13^, Nle^14^] BN (6–14) [37], [38] and the metallated panbombesin peptide [Y^III^-DOTA^0^, GABA^1^, D-Tyr^6^, βAla^11^, Thi^13^, Nle^14^] BN (6–14) [12]. Nock et al [9] developed a BN-based peptide with an *N*-terminal open-chain tetraamine framework ([(N_4_-Bzdig)^0^] BN (7–14)) for ^99 m^Tc-labeling, which has high affinity to NMB-R (0.65 nM) and GRP-R (0.9 nM) but negligible affinity to BNRS-3 (37 nM). Our previous published [^nat^Ga/^nat^Lu]-DOTA-PESIN (DOTA-PEG_4_-BN(7–14) [40] also showed moderate affinities to NMB-R (12.5 nM) and GRP-R (10.0 nM), and no affinity to BNRS-3 (>1000 nM). These results underscore the role of βAla^11^ as a key factor in maintaining high affinity to BNRS-3.

### Metabolic Stability

Compared to our previously developed radiopeptide, [^111^In]-BZH2 (^111^In-DOTA-[D-Tyr^6^, βAla^11^, Thi^13^, Nle^14^] BN(6–14)) [12], [^111^In]-BZH4 has a 3.5 times lower stability in serum, which indicates that the replacement of Leu^13^ by an unnatural amino acid (Thi) stabilizes the peptide significantly. This result also implies that the peptidases (CD10/NEP) [41] may be responsible for the cleavage of His^12^-Leu^13^ or His^12^-Thi^13^. In the prototype of [D-Tyr^6^, βAla^11^, Nle^14^] BN(6–14), DOTA-GABA and CPTA conjugated to the peptide have a similar stability. However, compared to ^nat^Cu-CPTA-D-Tyr-[βAla^11^] BN(7–14), both ^nat^Cu-CPTA and ^nat^Cu-CPTA-Gly conjugated-[βAla^11^] BN(7–14) analogues showed a 5-fold higher stability. Furthermore, Met^14^ ([^64/67^Cu]-BZH6) afforded two fold higher stability than Nle^14^ ([^64/67^Cu]-BZH5).

### *In vivo* Evaluation

Rapid internalization and efficient trapping allow for an optimal tumor-to-background ratio for imaging and are even more important for success of targeted radionuclide therapy. All [^64/67^Cu]-labeled BN analogs showed a specific uptake in PC-3 tumor and GRP receptor-positive organs such as pancreas. This was further illustrated through the decreased uptake of the probe in GRP-R expressing tissues when a cold blocking dose was co-administered. The [^64/67^Cu]-BZH7 showed the greatest uptake and long retention time in PC-3 tumors. This is likely due to its highest binding affinity and internalisation rate (among the tested compounds) and a relatively slow externalization rate. These results are comparatively better than those of negatively charged BN analogs such as [^64^Cu]-DOTA-Aoc-BN(7–14) [35] and [^64^Cu]-DOTA-[Lys^3^] BN [36]. As a result, [^64^Cu]-BZH7 has significant potential to perform as a high-resolution tool for clinical diagnosis, e.g. in the detection of micro-size metastases.

The uptake of all [^64^Cu-CPTA] BN analogs in liver and kidneys were high; which also held for the other [^64^Cu-DOTA]-conjugated BN analogs [35], [36]. High liver uptake of other CPTA-labeled peptides (octreotide) has previously been observed [29]. These results indicate that the charge of ^64^Cu-labeled BN (or ^64^Cu-CPTA/DOTA systems) has no influence on the excretion pathway. However, high liver accumulation of radioactivity in the case of [^64^Cu-DOTA]-conjugated BN analogs was attributed to possible *in vivo* demetallation of Cu-64 from DOTA [42]. Garrison et al. have shown that the cross-bridged cyclam based radioligand, [^64^Cu]-CB-TE2A-Aoc-BN(7–14) (CB-TE2A, 1,4,8,11-tetraazabicyclo[6.6.2]hexadecane-4,11-diacetic acid) exhibits lower liver uptake and improved *in vivo* stability compared to [^64^Cu]-DOTA-Aoc-BN(7–14) [42]. Recently, application of the triazacyclononane-based ligand framework showed high *in vivo* kinetic stability of the ^64^Cu^2+^ complex. The more compact ^64^Cu-NOTA complex of [^64^Cu]-NOTA-Aoc-BN(7–14) (NOTA, 1,4,7-triazacyclononane-1,4,7-triacetic acid) appears to overcome demetallation and uptake of radiometal by hepatobiliary proteins and accumulation and retention in renal tissue *in vivo* [43]. We have recently shown that NODAGA (1,4,7-triazacyclonane, 1,4-acetic acid, 7-glutaric acid) enables labeling with ^64^Cu at room temperature and high *in vivo* stability [44]. Another interesting Cu^2+^-chelator family is the macrobicyclic cage amine ligands, SarAr (1-N-(4-aminobenzyl)-3,6,10,13,16,19-hexaazabicyclo [6,6,6]-eicosane-1,8-diamine) that can form very stable Cu^2+^ complexes, and has recently been functionalized with bombesin analogs [45]. However, cyclam (1,4,8,11-tetraazacyclotetradecane) was chosen because it has been successfully used *in vivo* when coupled to MAbs [28]; in addition, the Pomper group showed excellent *in vivo* pharmacoknetics of ^64^Cu-labeled bis-cyclam analog for targeting CXCR4 receptor, such as ^64^Cu-AMD3465 [31]. These recent studies signify that BN analogs conjugated to cross-bridged (or macrobicylized) cyclam- or triazacyclononane- based chelators may be more preferable for radionuclide therapy with ^64/67^Cu. Therefore the CPTA-chelator may not be ideal when coupled to peptides despite the very high kinetic and thermodynamic stability of its Cu(II) complexes in vitro.

A hippurane-type molecule might facilitate clearance through kidney (as is generally preferred for radioligands), and the introduction of a glycine after CPTA leads to a hippurane-type spacer molecule. Therefore, this structural modification was expected to show similar behaviour. Our results (Figure 5) confirmed this hypothesis. Even when GRP receptor expressed in mice was blocked by an excess of [Cu^II^]-BZH7, [^64/67^Cu]-BZH8 was excreted faster from the kidney than [^64/67^Cu]-BZH7. This clearly indicates that the hippurane-type spacer molecule integrated in BN analogues hastens renal excretion of injected radioactivity. However, the tumor-to-kidney ratio of [^64/67^Cu]-BZH8 was not improved because this modification also resulted in a lower GRP receptor affinity and concomitantly a lower tumor uptake.

### Conclusion

In this work, we pursued a series of modifications of bombesin receptor targeting peptides in order to generate improved PET and therapeutic radioligands for pre- and clinical investigation. These studies show that charge at the *N*-terminus of radiometal labeled BN peptides has a significant influence on the rate of internalization and the binding affinity to human GRP receptor. Interestingly, this effect is substantially less significant for binding to mouse GRP receptor. This observation supports earlier findings that the careful selection of animal species and tumor origin is absolutely mandatory in order to evaluate new radioligands in general [46], and for the radiolabeled bombesin analogs in particular [27].

Pharmacokinetic considerations were also evaluated through the introduction of a hippurane-like spacer into a ^64/67^Cu-labeled BN analogue. This led to an improved kidney clearance. The replacement of Met^14^ by Nle^14^ lowered the binding affinity of BN analog to human GRP receptor, which might be a potential cause for the lower internalization rate, as well as decreased tumor and pancreas uptake. The ^64/67^Cu labeled BZH7 (CPTA-[βAla^11^] BN(7–14)) showed favorable qualities as a targeting vector, which suggest its potential for localization and treatment of GRP-receptor positive tumors. The relatively low *in vivo* stability of the Cu^II^-CPTA complexes may be improved by cross-bridging CPTA, a strategy which we are pursuing currently.
